# Wissen über humane Papillomaviren unter Studierenden in Deutschland: eine Querschnittstudie belegt dringenden Handlungsbedarf

**DOI:** 10.1007/s00120-024-02436-x

**Published:** 2024-08-29

**Authors:** Cem Aksoy, Laila Schneidewind, Marius Butea-Bocu, Philipp Reimold, Sandra Schönburg, Johannes Huber, Radu Alexa, Matthias Saar, Jennifer Kranz

**Affiliations:** 1 Klinik für Urologie, Philipps-Universität Marburg, 35033 Baldingerstraße, Deutschland; 2 Urologische Universitätsklinik, Universität Bern, Bern, Schweiz; 3 Urologisches Kompetenzzentrum für die Rehabilitation – UKR, Kliniken Hartenstein, Bad Wildungen, Deutschland; 4 Universitätsklinik und Poliklinik für Urologie, Universitätsklinikum Halle (Saale), Halle (Saale), Deutschland; 5 Klinik für Urologie und Kinderurologie, Uniklinik RWTH Aachen, Aachen, Deutschland

**Keywords:** Condylomata acuminata, Prävention, Impfrate, Peniskarzinom, Intimkontakt, Condylomata acuminata, Prevention and control, Vaccination rate, Penile cancer, Intimate contact

## Abstract

**Hintergrund und Fragestellung:**

Humane Papillomaviren (HPV) zählen zu den häufigsten durch Intimkontakte übertragenen Erregern und verursachen viele gut- und bösartige Erkrankungen. Eine Impfung gegen diese Viren schützt sehr sicher vor diesen Erkrankungen. Trotz einer durch die Ständige Impfkommission (STIKO) empfohlenen HPV-Impfung sind das Wissen und die Impfrate in Deutschland sehr niedrig. Ziel der Studie war es daher, das HPV-Wissen unter deutschen Studierenden zu erheben.

**Material und Methoden:**

Zwischen 06/2019 und 01/2024 wurde eine deutschlandweite Online-Umfrage über den HPV-Wissensstand unter Studierenden über die jeweiligen Fachschaften verteilt. Die Umfrage beinhaltete 2 Aspekte: 1) Grundcharakteristika der Teilnehmer und 2) Wissensfragen über HPV (z. B. Übertragungswege, Effektivität und Nebenwirkungen der Impfung, Wissen über die HPV-Subtypen). Die Datenerhebung erfolgte anonym.

**Ergebnisse:**

Insgesamt haben 459 Studierende an der Studie teilgenommen. Hiervon waren 335 (72,98 %) Frauen, 122 (26,57 %) Männer und 2 (0,45 %) haben keine Angabe über ihr Geschlecht gemacht. Das Durchschnittsalter betrug 24,02 Jahre und die meisten Teilnehmer befanden sich im 6. (23,31 %/*n* = 107) Semester. 75,82 % (*n* = 348) der Teilnehmer waren Medizinstudierende und am zweithäufigsten mit 19,61 % (*n* = 90) nahmen Studierende der Rechtswissenschaften teil. Der häufigste vertretene Studienort war Aachen mit 270 (58,82 %) Teilnehmern. Nur die Hälfte (48,80 %/*n* = 223) der Studierenden wusste, dass jährlich ca. 8000 neue Krebserkrankungen in Deutschland HPV-assoziiert sind. Bezüglich der HPV-Impfung wusste mehr als ein Drittel (35,82 %/*n* = 163) der Teilnehmer nicht, dass diese auch vor Genitalwarzen schützt, während 21,93 % (*n* = 100) der Teilnehmer nicht wussten, dass von der HPV-Impfung auch Jungen profitieren können und diese nicht nur vor Gebärmutterhalskrebs schützt.

**Diskussion:**

Trotz mehrjähriger HPV-Impfempfehlung durch die STIKO ist das Wissen über HPV unter Studierenden weiterhin sehr gering. Es bedarf zur Behebung der HPV-Wissenslücken weiterer Prävention- und Aufklärungsarbeit mit dem zusätzlichen Ziel, die HPV-Impfrate zu verbessern.

## Abstract (en)

**Background and objectives:**

The human papillomavirus (HPV) is one of the most common viruses transmitted through intimate contact, causing many benign and malignant diseases. Vaccination protects against these diseases. Despite the HPV vaccination being recommended by the Standing Committee on Vaccination (STIKO), knowledge and vaccination rates in Germany are very low. Therefore, the aim of the study was to assess the HPV knowledge among German students.

**Materials and methods:**

Between 06/2019 and 01/2024, a nationwide online survey on HPV knowledge among students in Germany was distributed via the respective student councils. The survey included two aspects: 1) basic characteristics of the participants and 2) knowledge questions about HPV (e.g., transmission routes, effectiveness, and side effects of vaccination, knowledge about the HPV subtypes). Data collection was anonymous.

**Results:**

A total of 459 students took part in the study. Of these, 335 (72.98%) were female, 122 (26.57%) were male, and 2 (0.45%) did not specify their gender. The average age of the students was 24.02 years, and most of the participants were in their 6th (23.31%/107) semester. In all, 75.82% (348) of the participants were medical students followed by law students with 19.61% (90) participants. The most represented study location was Aachen, with 270 (58.82%) participants. Only about half (48.80%/223) of the students knew that approximately 8000 new cancer cases in Germany are HPV-associated annually. Regarding the HPV vaccination, more than a third (35.82%/163) of participants did not know that it also protects against genital warts, while 21.93% (100) did not know that boys can also benefit from the HPV vaccination and that it does not only improve HPV protection of girls against cervical cancer.

**Conclusion:**

Despite several years of HPV vaccination recommendation by the Standing Committee on Vaccination (STIKO), knowledge about HPV among college students still remains very low. Further prevention and educational efforts are needed to address HPV knowledge gaps, with the additional goal of improving HPV vaccination rates.

## Hintergrund

Humane Papillomaviren (HPV) sind die Ursache für viele gut- und bösartige Erkrankungen. Gutartige Feigwarzen (Condylomata acuminata) werden von den Subtypen 6 oder 11 verursacht und durch die Infektion mit den Hochrisiko-HPV-Subtypen 16, 18, 31 oder 45 können unterschiedliche bösartige Tumoren ausgelöst werden. Beispiele für bösartige Erkrankungen sind neben Gebärmutter‑, Rachen- und Analkrebs auch das Peniskarzinom, von welchem ca. die Hälfte durch Hochrisiko-HPV verursacht werden [1, 2]. Den effektivsten Schutz vor einer HPV-Infektion stellt die HPV-Impfung vor dem ersten Geschlechtsverkehr dar. Dementsprechend kommt auch hier der Prävention eine äußerst wichtige Rolle zu [3, 4].

Abhängig vom Impfstoff, schützt dieser gegen bestimmte Niedrig- und Hochrisiko-HPV-Subtypen. Die Ständige Impfkommission (STIKO) empfiehlt daher seit 2007 die Impfung für Mädchen und seit 2018 auch für Jungen im Alter zwischen 9 und 14 Jahren [5]. In Deutschland ist jedoch das Wissen zu gesundheitsrelevanten Themen unter Jugendlichen sehr gering. Dies zeigte eine Studie der Ärztlichen Gesellschaft zur Gesundheitsförderung e. V. (ÄGGF) im Rahmen des Präventionsprojekts „wICHtig“. Hier wurde u. a. das Wissen über Masern, Rauchen, Geschlechtskrankheiten, Schwangerschaft und HPV an 6256 Schülerinnen und Schülern, an 55 Schulen in insgesamt 8 Bundesländern ab der 7. Klasse in allen gängigen Schulformen erhoben. Auch das ärztliche Informationsgespräch im Rahmen der Kampagne in den jeweiligen einzelnen Klassen der Schülerinnen und Schüler wurde hierbei ausgewertet.

Die Ergebnisse zeigten, dass das Wissen über gesundheits- und präventionsrelevante Themen unter den Jugendlichen sehr gering ist und dass sich dieses durch ärztliche Arbeit verbessern lässt. Interessanterweise wurde in der Studie ebenfalls die Bekanntheit der Begriffe „J1- und J2-Untersuchung“ erfragt, eine Vorsorgeuntersuchung für Jugendliche. Bei der ersten Befragung waren nur 24,4 % der Befragten über dieses Angebot informiert [6].

Eine andere Studie, die das Wissen über „Jungengesundheit“ an 7 Gymnasien in Nordrhein-Westfalen untersuchte, erbrachte unabhängig vom Wissensdefizit der Jugendlichen u. a. auch über Geschlechtskrankheiten, einen niedrigen Kenntnisstand über Themen der „Jungengesundheit“ sowie einen Wissensvorsprung von Schülerinnen von durchschnittlich 6 % mehr korrekter Antworten [7]. Diese Wissensdefizite stellen für unsere Gesellschaft sehr ernstzunehmende Gefährdungen dar. Daher ergibt sich daraus auch eine gesamtgesellschaftliche Aufgabe.

Als Maßnahme zur Steigerung der Wahrnehmung der Chancen der HPV-Impfung in der Bevölkerung führten die Deutsche Gesellschaft für Urologie e. V. (DGU), der Berufsverband der Deutschen Urologie e. V. (BvDU) und die ÄGGF im Jahre 2018 eine „Themenwoche HPV“ ein. Seither gibt es zu der Thematik auch ein HPV-Informationsportal für Jugendliche und Eltern im Internet (www.hpv-portal.de; [8]).

Trotz vieler Bemühungen von Fachgesellschaften und gemeinnützigen Vereinen ist das Wissen über viele relevante Gesundheitsthemen, insbesondere über HPV, in der Gesellschaft unterrepräsentiert [3, 9, 10]. Ziel dieser Arbeit war es daher, das Wissen über das Thema HPV unter Studierenden in Deutschland zu ermitteln.

## Methoden

Wir führten vom 01.06.2019 bis 01.01.2024 eine bundesweite Online-Umfrage zum Wissen über HPV durch, allerdings wurde der Einladungslink in diesem Zeitraum nur einmalig versandt. Die Umfrage beinhaltete 15 Fragen, welche sich in zwei Teilbereiche gliedern. Der erste Teil beinhaltet die Grundcharakteristika der Teilnehmer und der zweite das Wissen über HPV. Ermittelt wurden bei den Grundcharakteristika das Geschlecht, das Alter, der Studienort, der Studiengang und das Studiensemester. Die Wissensfragen im zweiten Teil beinhalteten die HPV-Übertragungswege, Effektivität und Nebenwirkungen der Impfung, Impfraten unter Mädchen, Inzidenz HPV-assoziierter Krebserkrankungen, die HPV-Impfempfehlung für Jungen, Wissen über die HPV-Subtypen und epidemiologisches Wissen zu HPV.

Der Fragebogen wurde mit dem Umfrage-Tool SurveyMonkey® (SurveyMonkey Inc., San Mateo, USA) erhoben und mit einem Einladungslink zur Online-Umfrage verteilt. Die Datenerhebung erfolgte anonym und der Fragebogen war nicht validiert. Über den E‑Mail-Verteiler unterschiedlicher Fachschaften an deutschen Universitäten wurden die Teilnehmer der Studie rekrutiert.

Eine Rückläuferquote konnte nicht ermittelt werden, da nicht kontrollierbare Dopplungen in den genutzten E‑Mail-Verteilern bestanden. Die kategorialen Variablen werden als absolute und relative Häufigkeiten berichtet und kontinuierlichen Variablen als Mittelwert, Median und Interquartilsabstand (IQR). Die deskriptiven statistischen Analysen wurden mit SPSS 26.0 (IBM, Corp., Armonk, NY) durchgeführt.

Eine ethische Genehmigung für diese Studie war aufgrund der Anonymität gemäß den lokalen und nationalen Richtlinien nicht erforderlich. Die Studie wurde in Übereinstimmung mit der Deklaration von Helsinki des Weltärztebundes durchgeführt.

## Ergebnisse

### Grundcharakteristika der Studierenden

Die Studie rekrutierte insgesamt 459 Teilnehmer mit einem Durchschnittsalter von etwa 24 Jahren und einem medianen Alter von 23 (IQR 22–25) Jahren. 73,30 % der Teilnehmer waren weiblich und 26,70 % männlich. Zum Geschlecht haben 2 (0,44 %) Teilnehmer keine Angaben gemacht. Die Mehrheit der Studierenden befand sich im dritten Studienjahr (*n* = 152; 33,26 %). Das 6. Studiensemester war mit 107 (23,41 %) Teilnehmern am häufigsten vertreten. Die meisten Studierenden waren mit 348 (75,82 %) aus der Humanmedizin, gefolgt von Studierenden der Rechtswissenschaften mit 90 (19,61 %). Insgesamt waren 9 Studienfächer in der Umfrage vertreten (Tab. 1). Aachen weist mit insgesamt 270 (58,82 %) die höchste Zahl an Teilnehmern der Umfrage auf und wird gefolgt von Halle mit 135 (29,41 %) Teilnehmern. Die geringste Zahl der Teilnehmer kam aus Bremen, Dortmund und Mainz mit jeweils nur einem (0,22 %) Teilnehmer (Tab. 1).

**Tab. 1:** Grundcharakteristika der Studierenden

	Kohorte (*n* = 459)
*Alter (Jahre* [Mittelwert; Median; IQR])	24,02; 23; 22–25
*Geschlecht* (*n* [%])	
Weiblich	335 (73,30)
Männlich	122 (26,70)
k. A.	2
*Universitätsstadt*	
Aachen	270 (58,82)
Bremen	1 (0,22)
Dortmund	1 (0,22)
Freiburg	9 (1,96)
Halle	135 (29,41)
Hamburg	42 (9,15)
Mainz	1 (0,22)
*Studienrichtung*	
Rechtswissenschaften	90 (19,61)
Humanmedizin	348 (75,82)
Zahnmedizin	1 (0,22)
Theologie	6 (1,31)
Lehramt	2 (0,44)
Soziologie	7 (1,53)
Heilpädagogik	1 (0,22)
Psychologie	1 (0,22)
Betriebswirtschaftslehre	3 (0,65)
*Studiensemester*	
1. Semester	2 (0,44)
2. Semester	12 (2,63)
3. Semester	11 (2,41)
4. Semester	19 (4,16)
5. Semester	45 (9,85)
6. Semester	107 (23,41)
7. Semester	26 (5,69)
8. Semester	60 (13,13)
9. Semester	31 (6,78)
10. Semester	77 (16,85)
11. Semester	24 (5,25)
12. Semester	43 (9,41)
k. A.	2

*IQR* Interquartilsrange, *k.* *A.* keine Angabe

### Allgemeines Wissen über HPV unter Studierenden

Die Antworten zu den allgemeinen HPV-Fragen, des HPV-Übertragungsweges sowie zu den HPV-Subtypen sind in Tab. 2 dargestellt. Zusammenfassend zeigte sich in der Umfrage ein hoher Wissenstand zu den HPV-Subtypen und den damit assoziierten Erkrankungen als auch zu den HPV-Übertragungswegen. Das HPV-Wissen zur Prävalenz und zu HPV-assoziierten Krebsfällen zeigte sich wiederum gering.

**Tab. 2:** Studienfragen und Antwortverteilung der Studierenden zum Thema humane Papillomaviren (HPV)

	Kohorte, *n* = 459
Wie viele Männer sind schätzungsweise mit dem humanen Papillomavirus infiziert?	
5–10 %	58 (12,69 %)
*15–20* *%*	*198 (43,33* *%)*
40–60 %	176 (38,51 %)
80–90 %	25 (5,47 %)
k. A.	2
Ist die folgende Aussage korrekt? Das humane Papillomavirus ist ein häufiges Virus, das die Haut und Schleimhäute befällt. Es gibt mehr als 150 verschiedene HPV-Typen. Etwa 30 HPV-Typen werden ausschließlich durch direkten Genitalkontakt übertragen. Diese „genitalen“ HPV-Typen sind entweder: 1) vom „Hochrisiko“-Typ – dies bedeutet, dass sie bestimmte Krebsarten verursachen können, oder 2) vom „Niedrigrisiko“-Typ – dies bedeutet, dass sie Genitalwarzen verursachen können	
*Ja*	*428 (93,86* *%)*
Nein	28 (6,14 %)
k. A.	3
Wie viele Krebsfälle pro Jahr in Deutschland lassen sich auf HPV zurückführen?	
*ca. 8000 Fälle*	*223 (48,86* *%)*
ca. 1000 Fälle	125 (27,35 %)
ca. 20.000 Fälle	98 (21,44 %)
ca. 200 Fälle	11 (2,41 %)
k. A.	2
Typischerweise werden die humanen Papillomaviren, die Genitalwarzen und Krebs verursachen können, folgendermaßen übertragen:	
*Beim Geschlechtsverkehr oder intimen Hautkontakt (Genitalkontakt)*	*446 (97,81* *%)*
Beim Einatmen von Luft	3 (0,66 %)
Beim Berühren von Gegenständen wie z. B. Türgriffen	5 (1,10 %)
Beim Händeschütteln	2 (0,44 %)
k. A.	3

*k.* *A.* keine Angabe, *kursiv* korrekte Antworten

### Wissen über die HPV-Impfung unter den Studierenden

Ungefähr die Hälfte der Teilnehmer (49,23 %/*n* = 225) antworteten, dass ca. 70 % der 15-jährigen Mädchen gegen HPV immunisiert sind. 81,76 % (*n* = 372) wussten, dass die STIKO seit Juni 2018 die HPV-Impfung für Jungen im Alter von 9–14 Jahren empfiehlt. 21,93 % (*n* = 100) der Teilnehmer sagten, dass die geimpften Jungen selber von der HPV-Impfung nicht profitieren, jedoch der Schutz bei Frauen vor Gebärmutterhalskrebs sich verbessert. Ungefähr zwei Drittel (64,18 %/*n* = 292) der Befragten gaben an, dass die HPV-Impfung gegen Genitalwarzen Schutz bietet.

Die Antworten zu den Fragen zur HPV-Impfung sind in Tab. 3 veranschaulicht. Zu den Nebenwirkungen der Impfung und dem Fakt, dass sowohl Jungen als auch Mädchen geimpft werden können lag ein vergleichsweise hoher Wissensstand vor. Weniger gut war die Rate der richtigen Antworten zu HPV-assoziierten Krebserkrankungen und zur Impfrate bei 15-jährigen Mädchen. Die Frage zu den HPV-assoziierten Karzinomen beantworteten nur etwas mehr als die Hälfte (55,16 %) der Befragten richtig. Die richtige Antwort zur Durchimpfungsrate unter 15-jährigen Mädchen wurde von weniger als der Hälfte der Teilnehmer (41,79 %) richtig beantwortet.

**Tab. 3:** Studienfragen und Antwortverteilung der Studierenden zur HPV-Impfung (humane Papillomaviren)

	Kohorte, *n* = 459
Ist die folgende Aussage korrekt? Die Schutzimpfung gegen krebserregende humane Papillomviren (HPV) wird seit Juni 2018 auch für Jungen (9- bis 14-Jahre) von der Ständigen Impfkommission empfohlen. Somit können zukünftig Mädchen und Jungen von der Impfung profitieren	
*Ja*	*372 (81,76* *%)*
Nein	83 (18,24 %)
k. A.	4
Ist die folgende Aussage korrekt? Die geimpften Jungen profitieren selber nicht von der HPV-Schutzimpfung, sondern der Schutz ungeimpfter Frauen vor Gebärmutterhalskrebs wird hierdurch verbessert	
Ja	100 (21,93 %)
*Nein*	*356 (78,07* *%)*
k. A.	3
Die HPV-Impfung für Jungen schützt vor bestimmten Krebsarten. Kreuze die richtige Aussage an:	
*Mund-Rachen-Krebs und Analkrebs*	*251 (55,16* *%)*
Analkrebs und Hodenkrebs	46 (10,11 %)
Mund-Rachen-Krebs und Prostatakrebs	47 (10,33 %)
Peniskrebs und Hodenkrebs	111 (24,40 %)
k. A.	4
Ist die folgende Aussage korrekt? Die HPV-Impfung bietet zukünftig Schutz vor den weitverbreiteten Genitalwarzen. Diese sind zwar nicht lebensbedrohlich, dafür aber hartnäckig und unangenehm	
*Ja*	*292 (64,18* *%)*
Nein	163 (35,82 %)
k. A.	4
Wie hoch ist die Durchimpfungsrate bei den 15-jährigen Mädchen ungefähr?	
ca. 5 %	28 (6,13 %)
*ca. 30* *%*	*191 (41,79* *%)*
ca. 70 %	225 (49,23 %)
ca. 95 %	13 (2,85 %)
k. A.	2
Ist die folgende Aussage korrekt? Allergische Reaktionen können neben chronischen Schmerzen und Kreislaufschwäche als Nebenwirkung einer Impfung auftreten. Die HPV-Schutzimpfung hat ein sehr hohes allergisches Potenzial im Vergleich zu anderen Schutzimpfungen und wird daher in der Bevölkerung schlecht angenommen	
Ja	84 (18,42 %)
Nein	372 (81,58 %)
k. A.	3

*k.* *A.* keine Angabe*, kursiv* korrekte Antworten, *HPV* humane Papillomaviren

## Diskussion

Das Wissen unter Studierenden zur HPV-Thematik ist sehr niedrig. Trotz der hohen Kenntnis über die Übertragungswege des Virus (*n* = 446/97,81 %) sowie die HPV-Subtypen (*n* = 428/93,86 %), sind weiterhin viele Wissenslücken unter den Studierenden insbesondere zur HPV-Impfung nachzuweisen. Als Beispiel hierfür ist zu nennen sind, dass 84 Teilnehmer (18,42 %) angaben, dass die HPV-Impfung ein hohes allergisches Potenzial im Vergleich zu anderen Schutzimpfungen hat und dass 163 Teilnehmer (35,82 %) nicht wussten, dass die HPV-Impfung auch gegen Genitalwarzen schützt.

### Das Wissen über HPV unter Jugendlichen ist gering

Ungefähr die Hälfte (48,80 %/223) der Studierenden wusste, dass ca. 8000 neue Krebsfälle in Deutschland HPV-assoziiert sind. Nur etwas mehr als die Hälfte (55,16 %/*n* = 213) wusste hingegen, dass HPV Mund‑, Rachen- und Analtumoren verursachen kann. Dieser mangelhafte HPV-Wissensstand spiegelt sich in unterschiedlichen Kohorten, sowohl national als auch international wider.

In einer Studie an bayrischen Schulen zum Wissen über sexuell übertragbare Krankheiten mit dem Fokus auf HPV untersuchten Rummel et al. [11] ein Kollektiv von 4100 Schülerinnen und Schüler im Alter zwischen 12 und 17 Jahren im Zeitraum von September 2018 bis Februar 2019. Die Wissensermittlung erfolgte mittels Fragebogen. Insgesamt wurden 3834 Fragebögen ausgewertet und es konnte gezeigt werden, dass die HPV-Thematik unter den bereits wenig gegenwärtigen sexuell übertragbaren Krankheiten noch unbekannter in der Wahrnehmung der Jugendlichen war. Des Weiteren waren mit 30,4 % weniger als ein Drittel der teilnehmenden Mädchen gegen HPV geimpft und 45,3 % der Mädchen waren sich über ihren HPV-Impfstatus nicht sicher. Interessanterweise gab es unter den Nicht-HPV-Geimpften und den Teilnehmenden mit unbekanntem HPV-Impfstatus mit zwei Drittel (66,1 %) ein großes Interesse an einer HPV-Impfung.

In einer weiteren Studie die eine hochselektionierten Gruppe von 974 deutschen Medizinstudierenden anonym zum HPV-Wissensstand und Impfstatus befragte, war das Wissen über HPV sowie der Impfrate trotz medizinischer Kompetenz sehr gering [12]. Als Beispiel hierfür ist zu nennen, dass lediglich 28 % der Befragten, das Lebenszeitrisiko von HPV kannten und dass nur ungefähr die Hälfte (54 %) wussten, dass die Krankenkassen abhängig vom Alter die Kosten der HPV-Impfung übernehmen. Im Geschlechtervergleich zeigten sich ebenfalls große Unterschiede. Hier wussten z. B. signifikant mehr Frauen die richtigen Antworten auf die Frage zum Impfalter (94 % vs. 83 %, *p* < 0,001). Dieser Unterschied zwischen den Geschlechtern zeigte sich ebenfalls in der Impfrate. Während 82 % der Teilnehmerinnen gegen HPV geimpft waren, betrug die Anzahl der geimpften Teilnehmer lediglich 24 %. Das weibliche Geschlecht, die Beschäftigung mit der HPV-Thematik und das Thematisieren von HPV im Studium waren signifikant mit einer HPV-Impfung assoziiert.

Im internationalen HPV-Wissensvergleich unter Studierenden sind die Ergebnisse aus den USA, der Türkei, Irland oder Italien sehr ähnlich mit den deutschen Daten [13–16].

### Deutschland liegt in der HPV-Impfrate sehr weit zurück

Unabhängig vom Wissensstand über HPV, stellt sich die Frage, wie viele Jugendliche in Deutschland gegen HPV geimpft sind und v. a. wie sich Deutschland im europäischen Vergleich bezüglich der HPV-Impfrate positioniert. Ungefähr jeder Zweite (49,23 %/*n* = 225) sagte in unserer Umfrage, dass ca. 70 % der 15-jährigen Mädchen in Deutschland gegen HPV immunisiert sind. Dies spiegelt jedoch nicht die Realität wider.

Eine Studie zur Gesundheit von Kindern und Jugendlichen in Deutschland (KIGGS) des Robert Koch-Instituts untersuchte neben vielen gesundheitlichen Aspekten auch die HPV-Impfquote unter Mädchen. Diese wurden zu zwei unterschiedlichen Zeiträumen ermittelt. Die erste Erhebung erfolgte zwischen Juni 2009 bis Juni 2012 (KIGGS-Welle 1) und die zweite von September 2014 bis August 2017 (KIGGS-Welle 2). Die Ergebnisse der KIGGS-Welle 2 zeigten, dass unter den 11- bis 17-jährigen Mädchen 31,4 % über eine vollständige HPV-Impfung berichteten und dass dieses Ergebnis im Vergleich zu den gemeldeten Zahlen vor fünf Jahren der KiGGS-Welle 1 stabil geblieben ist [17].

Betrachtet man die HPV-Impfquote der Jungen in Deutschland, so war laut einer Studie von Wähner et al. [18] nach der HPV-Impfempfehlung der STIKO im Jahr 2018 diese anfänglich mit den monatlichen Zahlen der HPV-Erstdosen der Mädchen vergleichbar, nahm jedoch von Jahr zu Jahr stetig ab. Am Ende des Beobachtungszeitraums im Dezember 2021 betrug die Anzahl der vollständigen HPV-Impfungen 15-jähriger Jungen nur 26,4 %. Die Autoren sehen als Grund für die zwischenzeitlich niedrige HPV-Impfrate bei Jungen einen großen Einfluss der COVID-19-Pandemie („coronavirus disease“ 2019 [18]).

Im europäischen Vergleich findet sich die Impfquote 15-jähriger Mädchen in Deutschland auf den letzten Plätzen. Die höchsten Impfquoten finden sich in Portugal (95 %), Norwegen (88 %) und Island (85 %). In Portugal besteht eine traditionell hohe Impfakzeptanz während es in Norwegen und Island schulbasierte Impfprogramme gibt. Mit 31 % findet sich Deutschland zwar noch vor Frankreich (24 %), belegt jedoch in der gesamten Einstufung einen schlechten Platz. Sowohl Deutschland als auch Frankreich verfügen über kein schulbasiertes Impfprogramm [19].

### Es bedarf neuer Konzepte zur Steigerung der HPV-Awareness und Verbesserung der Impfquote

Im europäischen Vergleich sind in Deutschland trotz mehrjähriger STIKO-Empfehlungen für die HPV-Impfung sowohl die Impfquote als auch das HPV-Wissen unter Jugendlichen sehr niedrig. Dies spiegelt sich auch in unserer Umfrage wider. 21,93 % der Teilnehmer waren der Meinung, dass Jungen von der HPV-Impfung selbst nicht profitieren, sondern nur ungeimpfte Frauen dadurch einen besseren Schutz gegen Gebärmutterhalskrebs haben. Trotz des hohen Bildungsgrades und einer sehr hohen Beteiligung von Medizinstudierenden (75,82 %) zeigt die untersuchte Kohorte eher schlechte Ergebnisse und damit verbunden einen deutlichen Handlungsbedarf.

Die Einführung eines Impfangebotes in Schulen wäre eine Möglichkeit, die Impfquote zu verbessern. In einer Studie von Takla et al. [20] wurde ein HPV-Impfprogramm im hessischen Landkreis Bergstraße wissenschaftlich begleitet und zeigte, dass im untersuchten Landkreis mehr jüngere Mädchen HPV-geimpft wurden als in anderen hessischen Landkreisen.

Ein weiterer Angriffspunkt zur Steigerung des HPV-Wissens und damit verbunden mit der HPV-Impfrate stellt die Förderung der Teilnahme an der J1-Vorsorgeuntersuchung dar. Bei der J1-Vorsorgeuntersuchung handelt es sich um eine Gesundheitsuntersuchung für Jugendliche in Deutschland, wobei die Inanspruchnahme sehr gering ist [23]. Studien belegen, dass ein ärztliches Beratungsgespräch eine wichtige Rolle bei HPV-Impfentscheidungen spielt [21]. Somit ergibt sich neben der HPV-Aufklärung auch noch die Möglichkeit für den Arzt, über andere wichtige gesundheits- und präventionsrelevante Themen aufzuklären.

Erinnerungs- und Einladungssysteme zeigten am Beispiel mehrerer Krankheitsbilder eine große Zufriedenheit der Teilnehmerinnen und Teilnehmer und einen großen Vorteil. Ein Beispiel hierfür stellt das Brustkrebs-Screening dar, bei welcher die Interventionsgruppe (Erinnerungssysteme) eine signifikant höhere Screening-Rate im Vergleich zur Kontrollgruppe aufwies [22, 23]. Diese Vorteile könnten sich ebenfalls bei Jugendlichen am Beispiel der HPV-Impfung widerspiegeln [24].

Ebenso bergen soziale Medien ein großes Potenzial, um Jugendliche zu erreichen. Thompson et al. [25] untersuchten neue Strategien bei Facebook zu möglichen Auswirkungen sozialer Medien auf die öffentliche Gesundheit im Vertrauen zur HPV-Impfung. In der Studie öffnete sich ein Pop-up mit Weiterleitung auf eine seriöse Internetseite zur HPV-Impfung, vor der eigentlichen Weiterleitung nach dem Klicken auf einen Link zur HPV-Thematik. Die Integration der Pop-ups wurde von den meisten Teilnehmern als nützlich und akzeptabel bewertet. Ein Lenkungsgremium bestehend aus Experten und Influencern könnte in Zusammenarbeit mit staatlichen Behörden wie z. B. der Bundeszentrale für gesundheitliche Aufklärung (BZgA) geschlechtsspezifische Informationen in ansprechender Gestaltung unter Verwendung zielgruppenadaptierter Elemente in Bezug auf Design und Wortwahl schaffen. Dieses könnte zusätzlich um jährliche Präventionskampagnen erweitert werden, um das Bewusstsein für HPV-assoziierte Erkrankungen in der Gesellschaft zu verbessern.

## Limitationen und Stärken

Die Umfrage rekrutierte eine fachübergreifende Stichprobe von deutschen Studierenden zur HPV-Thematik, welche jedoch nicht repräsentativ für ganz Deutschland und mehrheitlich aus Aachen, Halle und Hamburg vertreten sind. Ebenso konnten Aspekte der HPV-Thematisierung im Studium nicht bewertet werden, da jede medizinische Fakultät ihr Curriculum selbst gestaltet und Studierende anderer Studiengänge trotz der medizinischen Fachfremde zur HPV-Thematik belesen und informiert sein konnten. Die fehlende Rückläuferquote sowie die Nutzung eines nicht validierten Fragebogens stellen weitere Limitationen der Studie dar. Trotz dieser Einschränkungen ermöglicht die Arbeit bei einem fachübergreifenden Kollektiv einen Überblick zum HPV-Wissen unter Studierenden in Deutschland.

## Fazit für die Praxis

- Unsere Studie zeigt, dass das Wissen über humane Papillomaviren (HPV) unter Studierenden in Deutschland trotz des hohen Bildungsstandes sehr gering ist.
- Zur Verbesserung des HPV-Wissens und zur Steigerung der HPV-Impfraten bedarf es noch mehr gesellschaftlicher Präventions- und Aufklärungsarbeit.
- Auch staatliche Impfangebote (z. B. in Schulen) könnten die Barrieren zur Inanspruchnahme der HPV-Impfung verringern.

## Abkürzungen

ÄGGF: Ärztlichen Gesellschaft zur Gesundheitsförderung e. V.
BvDU: Berufsverband der Deutschen Urologie
BZgA: Bundeszentrale für gesundheitliche Aufklärung
DGU: Deutsche Gesellschaft für Urologie e. V.
HPV: Humane Papillomaviren
KIGGS: Studie zur Gesundheit von Kindern und Jugendlichen in Deutschland
PATE: Prävention und Aufklärung testikulärer Erkrankungen
STIKO: Ständige Impfkommission
